# Editorial Board Members’ Collection Series: Theory and Simulation of Nanostructures

**DOI:** 10.3390/nano16171093

**Published:** 2026-09-01

**Authors:** Sotirios Baskoutas

**Affiliations:** Department of Materials Science, University of Patras, 26504 Patras, Greece; bask@upatras.gr

## 1. Introduction and Current Landscape

Computational Materials Science is one of the most important tools that have contributed to the huge developments in modern Nanotechnology [1]. Theoretical frameworks and atomistic simulations, ranging from First-Principles Density Functional Theory (DFT) and Reactive Molecular Dynamics to continuum wave and field modeling, allow scientists to study nanoscale dynamics with spatial and temporal resolution [2,3].

Despite rapid advancements, significant knowledge gaps persist across several frontiers:

- Multiscale Dissipation and Transport: Capturing non-equilibrium transport, energy dissipation, and saturation phenomena in ultra-fast quantum and single-molecule electronics [4,5].
- Complex Reactive Interfaces: Proposing reaction pathways, thermal stability, and decomposition kinetics under extreme thermal or shock conditions in multi-component nanostructures [6].
- Defect and Topological Engineering: Quantifying how atomic-scale defects, edge states, and topological boundaries influence macro-scale optical, electronic, and magnetic properties [7].
- Nanofluidic and Nanophotonic Enhancement: Exploiting local field localization, bound states in the continuum, and thermoacoustic couplings for high-performance sensing and energy transduction [8].

This Special Issue, “Editorial Board Members’ Collection Series: Theory and Simulation of Nanostructures,” brought together 11 high-impact contributions [9,10,11,12,13,14,15,16,17,18,19] that directly address these fundamental gaps.

## 2. Summary of Key Contributions

The 11 articles published within this Special Issue can be broadly categorized into four interconnected thematic categories:

Theme A: Quantum Transport, Electronic Properties, and Nanodevices

- Resonant Tunneling Nanostructures: Vetrova et al. [9] present a non-saturating compact model for resonant tunneling nanostructures by replacing the Lorentzian transparency profile with an inverse square hyperbolic cosine function to eliminate non-physical current saturation in the negative differential conductivity region.
- Topologically Defective Arm-Chair Graphene Nanoribbons: Louis et al. [10] present screened PPP and SSH-like tight-binding models accurately match DFT predictions for topological states in 7/9-AGNR superlattices while correcting the errors of local Hubbard and unscreened PPP models.
- Quinone-Based Molecular Devices: Conrad et al. [11] used DFT, NEGF, and Landauer formalisms to analyze electronic current flow in pH-sensitive quinone-functionalized graphene nanoribbon devices. The results show that electrons flow mainly along nanoribbon edges, with pH-controlled oxidation states modulating current transport and molecular switching.
- Quantum Emitters Near Topological Insulator Nanoparticles: Thanopulos et al. [12] study the strong coupling dynamics of a quantum emitter in the presence of a topological insulator (Bi_2_Se_3_) spherical nanoparticle. Utilizing electromagnetic Green’s tensor methods, they report large Purcell factors and energy transfer enhancement.

Theme B: Thermoacoustics, Nanophotonics, and Metasurfaces

- Thermoacoustic Autofocusing in Carbon Nanotube (CNT) Films: Rong et al. [13] study thermoacoustic sound generation in CNT films in the presence of acoustic metasurfaces. Their multiphysics finite-element simulations reveal acoustic autofocusing.
- Underwater CNT Sponge Transducers: Qi et al. [14] explore the transient thermoacoustic characteristics and heat transfer performance of carbon nanotubes embedded in fluid matrix.
- Hollow Cuboid Metasurfaces with Near-Infrared Quasi-BIC Modes: Algorri et al. [15] design a hollow cuboid metasurface supporting Quasi-Bound States in the Continuum.

Theme C: Thermal Decomposition and Surface Interactions

- Thermal Decomposition of Core–Shell Nanoparticles: Sun et al. [16] utilize Molecular Dynamics simulations to study the thermal decomposition kinetics of core–shell nanoparticles.
- Volatile organic compounds on Doped Graphene Substrates: Chen et al. [17] use Density Functional Theory to study the electronic properties and adsorption thermodynamics of volatile organic compounds interacting with doped graphene substrates.
- Vitamin C Affinity to TiO_2_ Nanotubes: Ugolotti et al. [18] apply hybrid Density Functional Theory to study the adsorption mechanisms, binding affinity, and electronic structure changes of Vitamin C molecules on titania (TiO_2_) nanotube surfaces.

Theme D: Nanostructure Formation

- Nanoribbon Formation: Eskandari et al. [19] employ Molecular Dynamics to study the formation of carbon nanoribbons inside the carbon nanotube.
